# Descending Necrotizing Mediastinitis in Healthy Young Adults: The Fatal Consequence of the Delayed Help

**DOI:** 10.3390/reports7020040

**Published:** 2024-05-21

**Authors:** Petar Uchikov, Maria Kraeva, Krasimir Kraev, Bozhidar Hristov, Dzhevdet Chakarov, Nedzhat Ali, Chavdar Stefanov, Angelina Mollova-Kyosebekirova, Tihomir Tenchev, Snezhanka Dragusheva, Elizabet Dzhambazova, Bistra Dobreva-Yatseva

**Affiliations:** 1 Department of Special Surgery, Faculty of Medicine, Medical University of Plovdiv, 4000 Plovdiv, Bulgaria; puchikov@yahoo.com (P.U.); drnedzhatali@gmail.com (N.A.); tihomir.tenchev88@gmail.com (T.T.); 2 Department of Otorhynolaryngology, Medical Faculty, Medical University of Plovdiv, 4000 Plovdiv, Bulgaria; kraevamaria93@gmail.com; 3 Department of Propedeutics of Internal Diseases, Medical Faculty, Medical University of Plovdiv, 4000 Plovdiv, Bulgaria; 4 Section “Gastroenterology”, Second Department of Internal Diseases, Medical Faculty, Medical University of Plovdiv, 4000 Plovdiv, Bulgaria; hristov.bozhidar@abv.bg; 5 Department of Propedeutics of Surgical Diseases, Section of General Surgery, Faculty of Medicine, Medical University of Plovdiv, 4000 Plovdiv, Bulgaria; dchakarov@mail.bg; 6 Department of Anaesthesiology, Emergency and Intensive Care Medicine, Medical Faculty, Medical University of Plovdiv, Plovdiv 4000, Bulgaria; chavdar.stefanov@mu-plovdiv.bg; 7 Department of General and Clinical Pathology, Faculty of Medicine, Medical University of Plovdiv, 4000 Plovdiv, Bulgaria; angelinamollovakyosebekirova@gmail.com; 8 Department of Nursing Care, Faculty of Public Health, Medical University of Plovdiv, 4000 Plovdiv, Bulgaria; sdragusheva68@gmail.com; 9 Faculty of Public Health, Medical University of Plovdiv, 4000 Plovdiv, Bulgaria; betidzhambazova@gmail.com; 10 Section “Cardiology”, First Department of Internal Diseases, Medical Faculty, Medical University of Plovdiv, 4000 Plovdiv, Bulgaria; bistra.yatseva@mu-plovdiv.bg

**Keywords:** mediastinitis, young adults, deep neck infections, toothache, sepsis, multiorgan failure

## Abstract

Introduction: Descending necrotizing mediastinitis is one of the most lethal forms of acute mediastinitis. It originates from an odontogenic or deep neck infection, which descends to the mediastinum through the fascial planes. It is a rare condition, but mortality rates remain high, especially in the presence of comorbidities or predisposing factors. Delay in diagnosis has been shown to be one of the most important factors for the disease outcome. Therefore, early diagnosis and treatment by a multidisciplinary team are of utmost importance. Case series: Four healthy young males with descending necrotizing mediastinitis were treated at our institution. None of them had any comorbidities, but all of them waited between 3 and 4 days before seeking medical help. Upon their arrival at the hospital, in addition to the presence of a severe neck infection, the presence of mediastinitis was also found. Despite the timely surgical treatment of both the source of the infection and the mediastinitis, three of the four cases had a fatal outcome. Conclusion: We believe that the time factor is of greater importance for the outcome of acute descending mediastinitis than the factors of age and presence of accompanying diseases. Proper and rapid treatment by a multidisciplinary team is essential, even for young and healthy people.

## 1. Introduction

Acute mediastinitis is an inflammation of the tissues that surround the structures in the mediastinum. It was first described by Herman Boerhaave in 1724 [1]. This is an extremely severe condition, and, if left untreated, leads to significant mortality. One of the forms of acute mediastinitis is descending necrotizing mediastinitis (DNM), or suppurative mediastinitis. The first description of this condition was made by Pearse in 1938. It is an acute and life-threatening condition that originates from an infection in the structures of the neck, which eventually extends to the mediastinum [2]. The neck infections that could lead to such a complication are odontogenic, pharyngeal, and cervical infections. They are responsible for the origin of an abscess in the maxillary, parapharyngeal, and retropharyngeal spaces. The abscess could lead to cervical necrotizing fasciitis, which spreads to the mediastinum through well-established spaces. These are, namely, the pretracheal, the vascular visceral, and the retrovisceral/prevertebral spaces. This process is also facilitated by gravity and negative pressure entering the chest cavity [3,4,5]. Endo et al. [6] proposed a classification of the mediastinal infection routes on the basis of the degree of mediastinal extension. Type I refers to infection localized to the upper mediastinum above the carina; type IIA refers to infection extending to the lower anterior mediastinum; and type IIB refers to infection extending to both the anterior and posterior mediastinum. The leading cause of the development of mediastinitis in the largest percentage of cases appears to be odontogenic infection [7].

The diagnostic criteria for DNM were presented in 1983 by Estrera et al. [8] and have been well established since then: 1—clinical manifestations of severe infection, 2—demonstration of characteristic radiographic findings, 3—documentation of necrotizing mediastinal infection at operation; and 4—establishment of the relationship of oropharyngeal or cervical infection with the development of the necrotizing mediastinal process. The clinical symptoms are diverse, but point to the beginning and progressive deepening of a serious condition. The clinical manifestation usually starts with the typical symptoms of the main process. These are often pain, fever, swallowing disorders, and speaking difficulties. If left untreated or not treated properly, the symptoms rapidly progress to dyspnea, tachypnea, pain in the area behind the sternum, chills, and a very high fever [9]. The deepening of the inflammation leads to the development of tissue edema with effusions and necrosis. The clinical presentation is marked by the development of sepsis and multiorgan failure [4].

The preferred modality to set the final diagnosis from the imaging studies is considered to be the CT scan of the head, neck, and chest. It is indicative of confirming the diagnosis and also of giving important information regarding not only the extent, but also the severity of the mediastinal involvement [10]. The treatment consists of a combination of empiric antimicrobial therapy and an optimal surgical drainage approach. Despite the measures taken, the mortality rate remains between 12.5% and 37.5% [11].

The presence of comorbidities or predisposing factors has been proven to facilitate the descent of the infection from the neck due to the worsened condition of the immune system and has been proven to worsen the patient’s prognosis despite the treatment measures carried out. Most often, these are diabetes mellitus, kidney failure, neutropenia, or alcohol abuse [12]. Age >55 years is also considered a predisposing factor for the development of a complication and progression to DNM [11]. Early diagnosis is fundamental for a good outcome from this serious and life-threatening condition. A delayed diagnosis leads to a fulminant course, which increases mortality rates [13].

## 2. Case Series Presentation

During the years 2018–2021, four young males with no comorbidities were admitted to our hospital with a severe infection involving the neck. One of the patients reported a severe toothache at the onset of the symptoms. All of them waited 3 to 4 days before seeking medical help. Upon arrival at the hospital, all patients had evidence of a neck infection, the spread of inflammation, and involvement of the chest cavity. All of them developed mediastinitis. The basic parameters of the patients’ history can be seen in Table 1. Radiological images and types of surgical treatment are also reviewed in Table 2. All four patients met the Estrera criteria for DNM. All of them had signs of severe infections, including fever, chest pain, and increased markers of inflammation. Typical radiographic findings with evidence for mediastinal widening and pus collection were also present. In all of them, the inflammation originated from a source located in the head and neck area. Evidence of the spread of inflammation to the mediastinum was found in the subsequent operative interventions. Drainage of pus from the mediastinum was performed. Damaged necrotic tissue was also removed.

**Figure 1:**
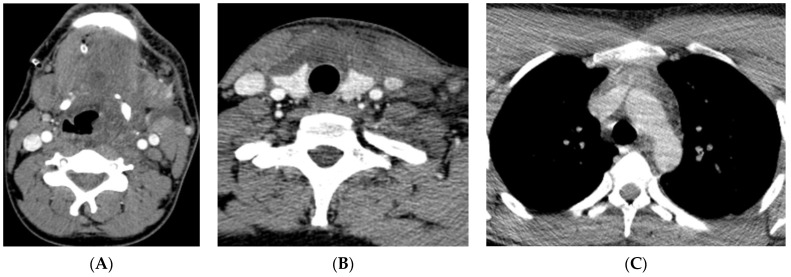
(Patient 1) Axial contrast-enhanced CT image; (**A**) abscess/hypodense lesions/in the left submandibular, parapharyngeal, pharyngeal mucosal and retropharyngeal spaces. (**B**) Mediastinitis in the visceral space. (**C**) Upper anterior mediastinum—mediastinitis.

**Figure 2:**
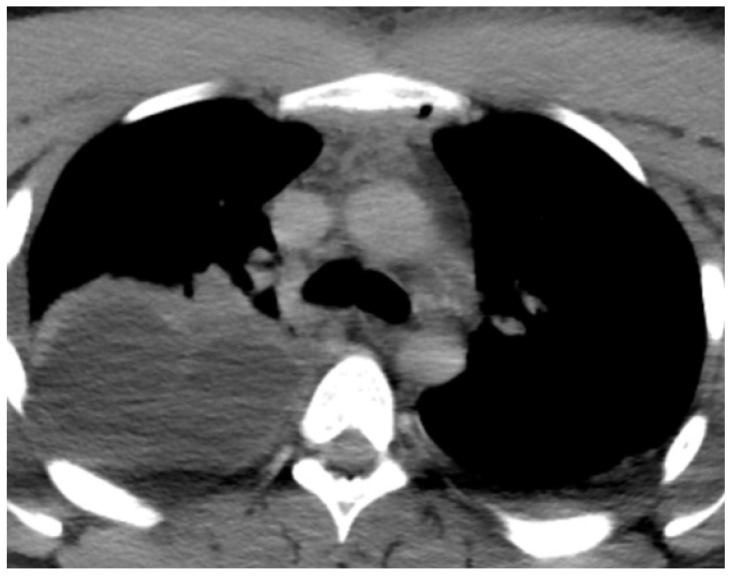
(Patient 2) CT angiography; upper anterior mediastinitis and right pleural effusion.

**Figure 3:**
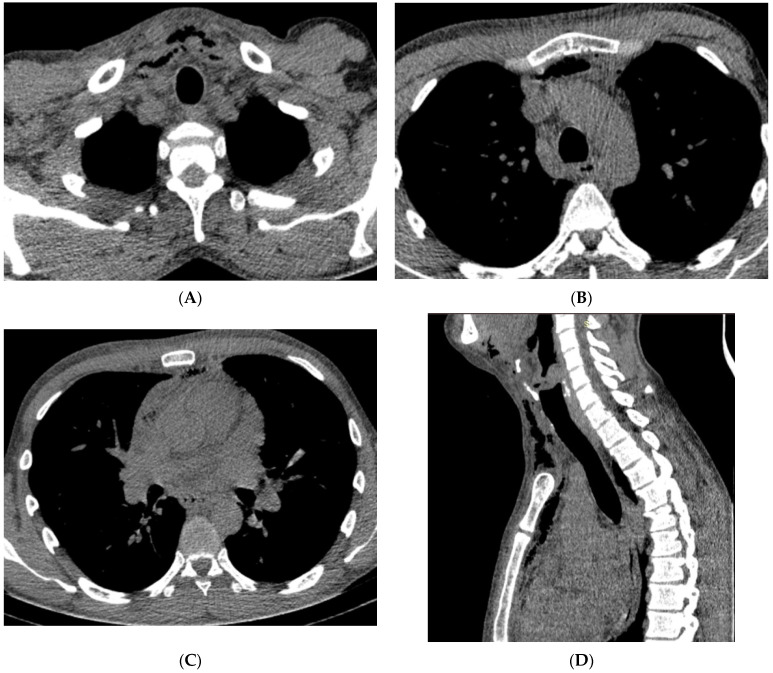
(Patient 3) Axial contrast-enhanced CT image; (**A**) air collection in the costoclavicular space, (**B**,**C**) air collection in the subclavian space, (**D**) air collection in the anterior mediastinum.

**Figure 4:**
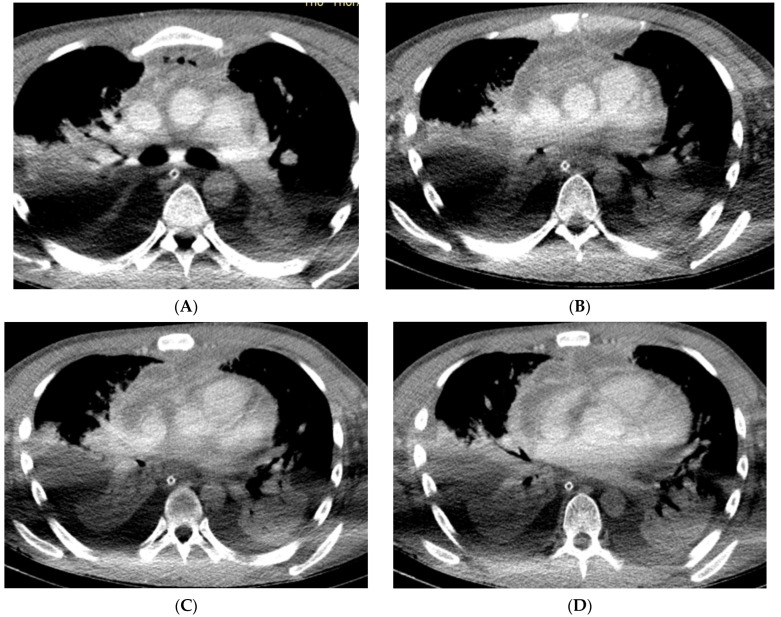
(Patient 4) Axial contrast-enhanced CT image; (**A**–**D**) mediastinal empyema on the anterior mediastinum and retrosternal space and bilateral pleural effusions.

These patients were four young men aged between 23 and 33 years. None of them had any comorbid conditions. In one of the cases, the initial infection started with an inflamed and suppurated tooth from the lower dentition on the left and gradually spread to the submandibular region. In the remaining three, the infection originated in the submandibular space without evidence of a dental infection. All experienced delayed diagnosis and treatment due to waiting 3 to 4 days prior to seeking medical help. The mean time between the onset of the symptoms and the development of mediastinitis was 3 days and 12 h (3 to 4 days). The main presenting symptoms were submandibular and neck pain, swelling, and fever. The blood tests of the patients are shown in Table 3. An urgent CT scan of the head, neck, and chest cavity was performed on all of the patients. Imaging studies showed the presence of a purulent collection starting from the tissues of the neck in the area of the submandibular space, covering the parapharyngeal space, and descending to the mediastinum, involving various compartments. The main imaging features were abscess formation, soft tissue thickening, and lymphadenopathy (Figure 1, Figure 2, Figure 3 and Figure 4).

All patients underwent emergency surgical treatment. The surgical management of these cases was conducted by a multidisciplinary team. First, multiple intra- and extraoral incisions were performed. Pus and necrotic tissue were removed, and the area was cleaned by irrigation. In one of the patients, the suppurated tooth was also extracted, and the area around it was meticulously cleaned. A submandibular incision parallel to the edge of the mandible was made, and the submandibular and parapharyngeal spaces were opened in all of the cases. Cleaning with irrigation was carried out, and all of the necrotic tissues and debris were removed. Irrigation with antiseptic solutions was also performed. The second part of the operative intervention was aimed at the severe chest infection. All patients underwent mediastinal drainage: three through suprasternal mediastinal drainage and one through thoracotomy, depending on the mediastinal compartments involved. A microbiology exam of the pus specimens showed that Staphylococcus aureus was the main pathogen in three of the patients and Acinobacter baumanii in one. Empiric antimicrobial therapy was also added. All four patients were referred to the ICU after the surgery.

The ICU stay in three of the cases was complicated by sepsis and multiorgan failure refractory to the ongoing treatment (Table 4). Despite the measures taken, these patients ended up with a fatal outcome. All of them underwent autopsies. The fourth patient survived. The autopsies of the three cases reviewed had similar macro- and microscopical findings. The serosal surfaces in the mediastinum appeared full of dark friable areas, diffusely covered by yellow-tan purulent and hemorrhagic material. The diagnosis was confirmed by the histology, which showed severe fascial necrosis with dense neutrophil infiltration and areas of hemorrhages.

The mean time between hospital admission and death was 14 days (6–25). The hospital stay of the survived patient was 30 days.

## 3. Discussion

Descending necrotizing mediastinitis is a type of acute mediastinitis that develops as a result of a deep neck infection. The complex anatomy in the areas of the gingiva, neck, pharyngeal space, and mediastinum facilitates the easy transition of the inflammatory process from one area to another due to the extensive fascial communication between these spaces. Descending of the infection is facilitated by gravity, negative intrathoracic pressure, and respiration [12].

Sada Urmeneta et al. describe the DNM as “one of the most lethal forms” of mediastinitis [14].

According to literature sources, the incidence of DNM due to a deep neck infection is 1.5–3.6% [15,16]. As assumed by Sugio et al., DNM is a disease that is relatively rare, and therefore physicians are often unexperienced with the treatment [3].

The origin of the deep neck infection could be any structure in the oropharyngeal and cervical areas. Based on the study of Ma et al., odontogenic infection plays a significant role in the development of deep neck infections [13]. Wheatley et al. also conclude that odontogenic infection is the most common oropharyngeal infection with mandibular second or third molar abscesses [17]. In a retrospective study performed by Qu et al., it was concluded that tonsil infections are more dangerous than all other maxillofacial infections because they lead more easily to parapharyngeal infection and eventually the formation of a neck abscess [18]. Dzian et al. report a case of a patient with purulent arthritis of the left sternoclavicular joint who has developed DNM [19].

The types of symptoms are determined by the initial infection. Most of the early symptoms are toothaches, sore throats, or fevers. These symptoms are followed by pain and swelling in the area of the mandible or neck, dysphagia, and dyspnea [4]. According to Pilav et al., the symptoms of mediastinitis are present between 12 h and 2 weeks after the start of the initial symptoms, but most often the symptoms of the DNM are present within the first 24 h [20]. In our cases, the main initial symptoms were pain and swelling in the submandibular and neck areas. These initial complaints were quickly followed by difficulty swallowing and difficulty breathing.

The diagnosis of DNM is based on the clinical presentation and typical CT findings. A CT scan is useful not only for the diagnosis, but also for the evaluation of the effectiveness of the therapy [18]. Benedetto et al. recommend that a CT scan be performed routinely every 48 h until the disease improves [21]. DNM was classified by Endo et al. according to its anatomical extent. Type I refers to infection localized to the upper mediastinum above the carina; type IIA refers to infection extending to the lower anterior mediastinum; and type IIB refers to infection extending to both the anterior and posterior mediastinum [6]. In three of our cases, the mediastinal extent of the inflammation refers to type I, and in one case, the mediastinal extent refers to type II.

As indicated by Ridder et al., the initial treatment of DNM includes intravenous antibiotics, airway management, treatment of the primary infection, and drainage of the neck and mediastinum [22]. Prado-Calleros et al. strongly recommend a multidisciplinary approach in the treatment of the disease [23]. In all of our cases, incisions and drainage of both the source of the infection and the mediastinum were performed. In one of the cases, a suppurated tooth was extracted from the lower dentition. Empiric antibiotic treatment was started according to an antibiogram. Postoperatively, the patients were transferred to the ICU, and follow-up scans were performed every 48 h.

Accompanying diseases play a significant role in the development of DNM. They also lead to an increase in the lethality of the disease. As stated by Kim et al., diabetes mellitus, chronic renal failure, and cardiovascular and pulmonary diseases are chronic conditions that play a significant role as risk factors [24]. According to Ma and colleagues, a diagnosis delay plays a very important role in the high mortality rates because of the rapid development of DNM [13].

We report four cases of deep necrotizing mediastinitis due to a deep neck infection. One of them had an odontogenic origin, and three of them originated from the submandibular area. All of the patients were young and healthy males with no comorbidities. The only predisposing factor in all of the patients was diagnosis delay due to the late seeking of medical help. Despite prompt operative treatment, antibiotic therapy, and postoperative care, three out of four patients had a fatal outcome.

We believe that the predisposing factor of time delay is more important than the presence of comorbidities.

All of the cases described involve young men without accompanying diseases. Timely surgical and conservative treatments were performed in all cases according to the sensitivity of the isolated causative agent. Three of the described cases resulted in fatalities, while the fourth individual survived. The virulence of the infection and the patient’s immune response are decisive factors. A faster and better response to the applied conservative treatment was observed in the surviving patient. It is believed that the individual immune response plays a crucial role in determining the disease’s outcome.

## 4. Conclusions

Descending necrotizing mediastinitis is a rare and severe life-threatening condition. It originates from infections of the neck and descends to the mediastinum. This complication must be detected as soon as possible. The diagnosis relies on a clinical picture and a CT scan. The treatment requires a multidisciplinary approach and includes antibiotic treatment, treatment of the infection source, and drainage of the neck and mediastinum. Risk factors for the development of DNM are diabetes mellitus, renal failure, and cardiovascular and pulmonary diseases. Predisposing factors are considered to be time delay and inadequate treatment. In our four cases, the delay in seeking medical help was more impactful compared to the lack of comorbidities. It can be concluded that early diagnosis and proper treatment are of utmost importance for the outcome of the disease.

## Figures and Tables

**Table 1:** Symptoms at the onset of the disease and comorbidities.

Patient	1	2	3	4
Sex/age	23/male	31/male	32/male	33/male
Toothache	-	-	-	+ *
Submandibular pain	+ *	+ *	+ *	+
Submandibular swelling	+	+	+	+
Neck pain	+	+	+	+
Neck swelling	+	+	+	+
Difficulty swallowing	+	-	+	-
Difficulty breathing	+	-	-	+
Fever	+	+	+	+
Diabetes mellitus	-	-	-	-
Kidney failure	-	-	-	-
HIV	-	-	-	-

* Chief complaint.

**Table 2:** Radiographic findings and surgical treatment.

Patient	1	2	3	4
Neck CT	Pus collection - in the left submandibular area - around the hyoid bone - around the left piriform recessus - around the thyroid cartilage - around the sternocleidomastoid muscle	Presence of a gas collection with delamination of the soft tissues of the neck in the area of the parapharyngeal space on the left Gas collections in - both palatine tonsils - anterior cervical space - left carotid space up to the level of the aortic arch - bilateral submandibular and sublingual space	Multiple air collections that stratify the tissues of the neck on the right side	Multiple air collections that stratify the superficial and deep tissues of the neck and enter the mediastinum
Chest CT	pus collection: ventral from the thyroid gland measuring 68/14 mm, with a density of 34–40 HU reaching the carina	Purulent collection starting from the neck and reaching the anterior mediastinum above the carina, presence of gas collections	Multiple air collections involving all departments of the anterior mediastinum above the carina	Massive pneumomediastinum, presence of fluid-purulent collections in the anterior and posterior lower mediastinum below the carina
Surgery	remediating the source of infection and suprasternal mediastinal drainage	Remediating the source of infection and suprasternal mediastinal drainage	Remediating the source of infection and suprasternal mediastinal drainage	Remediating the source of infection and thoracotomy

**Table 3:** Patients’ laboratory tests.

Patient	Hemoglobin (120–160) g/L *	Red Blood Cells (3.9–5.3) 10^12^/L *	White Blood Cells (3.5–10.5) 10^9^ g/L *	Platelets (140–400) 10^9^ L *	Fibrinogen (2.0–4.5) g/L *	C-Reactive Protein (0–10 mg/L) *	Erythrocyte Sedimentation Rate (2–25 mm/h) *
1	156	4.93	17.7 ↑	170	8.01 ↑	113.0 ↑	33 ↑
2	124	3.63 ↓	24.99 ↑	265.0	6.81 ↑	327.0 ↑	70 ↑
3	110 ↓	3.2 ↓	1.7 ↓	40 ↓	7.39 ↑	205.0 ↑	125 ↑
4	163 ↑	5.67 ↑	4.98	139.0	7.89 ↑	362.0 ↑	201 ↑

* Reference values; ↑ increased value; ↓ decreased value.

**Table 4:** ICU parameters.

Patient	1	2	3	4
FiO2	0.4	0.4	0.5	0.5
PEEP	8.5 cm H2O	8 cm H2O	5 cm H2O	8 cm H2O
PS	14 cm H2O	13 cm H2O	10 cm H2O	9 cm H2O
MV	10.5 L/min	10.5 L/min	13 L/min	13 L/min

FiO2: fraction of inspired oxygen; PEEP: positive end-expiratory pressure; PS: pressure support; MV: mechanical ventilation.

## Data Availability

Data available on request due to privacy restrictions.
